# Assessment of Exhaled Breath Condensate for *ALK* , *RET* , *ROS1* , and *NTRK1* Fusion Transcript Detection in NSCLC: Comparison With Tissue and Liquid Biopsy Samples

**DOI:** 10.1111/1759-7714.15513

**Published:** 2025-01-14

**Authors:** Aslı Tetik Vardarlı, Haydar Soydaner Karakus, Korcan Korba, Burcu Boluk, Su Ozgur, Cumhur Gunduz, Fusun Pelit, Ali Veral, Tuncay Goksel

**Affiliations:** ^1^ Ege University Faculty of Medicine Department of Medical Biology İzmir Turkey; ^2^ EgeSAM‐Ege University Translational Pulmonary Research Center Izmir Türkiye; ^3^ Ege University Faculty of Medicine Department of Pulmonary Medicine İzmir Turkey; ^4^ Ege University Faculty of Engineering Department of Chemical Engineering İzmir Turkey; ^5^ Regional Hub for Cancer Registration in Northern Africa Central and Western Asia, WHO/IARC‐GICR Izmir Türkiye; ^6^ Ege University Faculty of Science Department of Chemistry İzmir Turkey; ^7^ Ege University Faculty of Medicine Department of Pathology İzmir Turkey

**Keywords:** *ALK*, drug resistance, exhaled breath condensate (EBC), fusion transcripts, lung cancer, noninvasive, *NTRK1*, *RET*, RNA‐seq, *ROS1*

## Abstract

**Background:**

Lung cancer continues to be the primary cause of cancer‐related deaths globally, with the majority of cases identified at advanced stages. Genetic alterations, including mutations and gene fusions, are central to its molecular pathogenesis. The discovery of therapeutically targetable gene fusions, such as *ALK*, *RET*, *ROS1*, and *NTRK1*, has significantly advanced lung cancer management. Conventional methods, such as tissue biopsies, are invasive and unsuitable for continuous molecular monitoring. Consequently, noninvasive approaches, such as liquid biopsies and exhaled breath condensate (EBC), offer promising options for real‐time molecular surveillance.

**Methods:**

This study evaluates the feasibility of identifying fusion transcripts in 30 patients with lung adenocarcinoma by using next‐generation sequencing (NGS) on formalin‐fixed paraffin‐embedded (FFPE) tissue, plasma, and EBC samples.

**Results:**

Clinically significant fusion transcripts, including *KIF5B‐ALK*, *KIF5B‐RET*, and *SQSTM1‐ALK*, were detected across different sample types. EBC samples showed strong concordance with tissue biopsy results, particularly in detecting *ALK*, *ROS1*, and *RET* fusions, and demonstrated greater sensitivity than plasma in detecting *NTRK1* fusions. Additionally, 30 fusion transcripts of uncertain clinical significance were identified, highlighting the need for further research into their role in lung cancer pathogenesis.

**Conclusion:**

In conclusion, EBC samples provide a valuable, noninvasive medium for detecting clinically relevant and previously uncharacterized fusion transcripts in non‐small cell lung cancer (NSCLC). The high concordance between EBC and tissue biopsies suggests that EBC could complement tissue biopsy for effective diagnosis and monitoring of NSCLC. These findings underscore the importance of comprehensive molecular profiling using multiple sample types to enhance diagnostic precision and optimize therapeutic outcomes in lung cancer management.

## Graphical abstract

This study involved 30 Stage IIIB or IV lung adenocarcinoma patients from Ege University Medical School (2021–2023). Formalin‐fixed paraffin‐embedded (FFPE) tissue, plasma, and exhaled breath condensate (EBC) samples were collected for analysis, focusing on ALK, ROS1, RET, and NTRK1/2/3 fusion transcripts via next‐generation sequencing (NGS). Clinically significant fusions detected included KIF5B‐ALK, KIF5B‐RET, and SQSTM1‐ALK across various sample types. Notably, EBC samples showed strong concordance with tissue results, particularly for ALK, ROS1, and RET fusions, and outperformed plasma in NTRK1 detection, supporting EBC's potential as a noninvasive diagnostic tool.

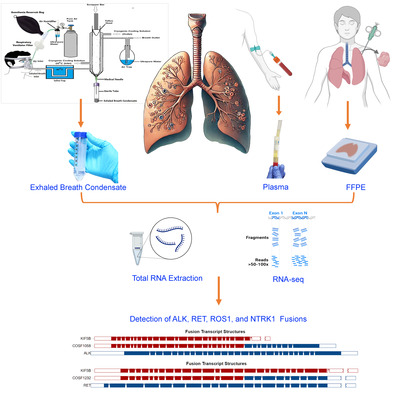

## 1 Introduction

Lung cancer (LC) continues to be the primary cause of cancer‐related deaths globally, with the majority of cases identified at advanced stages. According to GLOBOCAN 2020 statistics, LC represents 17.6% of all cancer cases worldwide [1]. In Turkey, 2020 data indicated 41 000 new LC cases, with age‐adjusted incidence rates of 41.7 per 100 000 in men and 8.7 per 100 000 in women [2]. In 2022, data from the Turkish Ministry of Health reported LC as the most common cancer type among men and the fifth most common among women, with approximately 30 000 new cases diagnosed annually. Non‐small cell lung cancer (NSCLC) is the predominant subtype, accounting for about 80% of all LC cases. Adenocarcinoma, a subtype of NSCLC, comprises around 40% of these cases, making it the most frequently occurring subtype of LC [3].

The advent of personalized medicine has transformed the diagnosis and management of LC by allowing clinicians to tailor therapies based on the unique histological and genetic profiles of a patient's tumor. By targeting specific molecular alterations, such as EGFR mutations, *ALK* rearrangements, and *ROS1* fusions, clinicians can implement more effective and precise treatment strategies. Advances in understanding the molecular pathogenesis of LC have enabled the identification of critical driver mutations and gene rearrangements that contribute to LC progression, thereby guiding the development of targeted therapies. NSCLC is now classified into actionable molecular subgroups based on genetic alterations that occur during epithelial cell transformation, allowing for optimized treatment plans tailored to these specific changes [4].

The identification of gene mutations, fusions, and rearrangements in NSCLC that can be targeted therapeutically has led to the development of treatments specifically designed to inhibit the oncogenic effects of these altered proteins. Beyond established targets like EGFR mutations and *ALK* gene fusions, other alterations, such as *ROS1* gene fusions, are increasingly recognized as predictive biomarkers, thereby broadening the range of patients eligible for targeted LC therapies [5]. Molecular alterations often activate oncogenes, including *EGFR*, *KRAS*, *NRAS*, *BRAF*, and *ERBB2*, primarily through point mutations and small insertions or deletions (indels). Additionally, chromosomal rearrangements involving tyrosine kinase receptor genes, such as *ALK*, *ROS1*, *RET*, and *NTRK1*, contribute significantly to NSCLC carcinogenesis, further diversifying the molecular landscape. These genetic rearrangements produce chimeric fusion kinases—aberrant proteins that drive oncogenic transformation and induce oncogene dependence in tumor cells. The prevalence of individual genetic rearrangements in NSCLC ranges from 1% to 7%, with a combined prevalence of all such events estimated at 5% to 9% [6]. Notably, *ROS1* and *RET* rearrangements occur in approximately 1% and 2% of lung adenocarcinoma cases, respectively [7].

Discovered in 2007, the *EML4*‐*ALK* fusion has since been a focus of research for its role in the molecular pathogenesis of LC and its applications in targeted therapy. *ALK* kinase inhibitors, such as crizotinib, are now established as the standard first‐line therapy for *ALK*–rearranged lung adenocarcinoma, offering superior progression‐free survival compared to conventional chemotherapy. Consequently, precise detection of *ALK* rearrangements is essential in molecular diagnostics to guide targeted treatment and improve patient outcomes. Among the Caucasian population, *ALK* rearrangements are present in approximately 3%–7% of lung adenocarcinoma cases [8]. Clinically approved tyrosine kinase inhibitors (TKIs) that target *ALK* fusion proteins include crizotinib, alectinib, and ceritinib, with crizotinib also demonstrating efficacy against *ROS1* rearrangements [9]. The advancement of fusion protein‐targeting therapies has highlighted the need for accurate, systematic, and preferably noninvasive diagnostic techniques to facilitate effective treatment selection.

The increasing use of targeted therapies for *ALK*, *ROS1*, and *RET* gene rearrangements has underscored the critical importance of routine genetic screening in patients with lung adenocarcinoma. According to the 2024 National Comprehensive Cancer Network (NCCN) guidelines, molecular testing is recommended for all NSCLC patients to detect *EGFR* and *BRAF* mutations, *ALK* and *ROS1* rearrangements, and PD‐L1 expression, without exception based on clinical characteristics. With continuous advancements in molecular biology, additional driver mutations and related therapies are rapidly emerging. Consequently, the effective diagnosis and management of LC now require the analysis of multiple driver mutations and fusion transcripts. Initial tumor biopsies taken at diagnosis often prove insufficient for repeated molecular assessments and may not capture the disease's evolving molecular profile. Therefore, noninvasive methods are essential for monitoring the dynamic molecular landscape throughout treatment and follow‐up [10].

Liquid biopsy, a primary noninvasive technique, analyzes cell‐free nucleic acids such as DNA (cfDNA) and RNA (cfRNA) circulating in extracellular fluids. Among these, circulating tumor‐derived DNA (ctDNA)—a tumor‐derived subset of cfDNA—has shown substantial promise in cancer management, enabling the detection of specific genetic alterations that inform targeted therapies, thus serving as an essential tool in precision oncology. Currently, ctDNA is widely applied in various clinical settings, including assessing tumor burden, identifying resistance mechanisms, and detecting mutations when tissue biopsies are unavailable. Circulating tumor‐derived RNAs have also been detected in blood, plasma, and serum from patients with multiple cancers, including pancreatic, gastric, nasopharyngeal cancers, and melanoma. Although RNA is generally considered more prone to degradation due to RNases, plasma RNA exhibits unexpected stability, offering new opportunities for noninvasive cancer diagnostics. Analyzing circulating RNA provides valuable insights into tumor biology, enhancing cancer management. As liquid biopsy technologies advance rapidly, they are expected to reshape oncology by enabling personalized treatment strategies and improving patient monitoring [11]. Noninvasive approaches, including liquid biopsies, have become integral to LC diagnosis and monitoring due to their capacity to detect ctDNA and other biomarkers. However, liquid biopsy techniques can be limited by variability in ctDNA abundance, which can be influenced by tumor location, burden, and the release of ctDNA into the bloodstream. Moreover, ctDNA can sometimes be present at levels below the detection threshold, particularly in early‐stage cancers or in cases with low tumor burden [12].

Exhaled breath condensate (EBC) analysis is emerging as a promising noninvasive method for the real‐time detection of molecular alterations in NSCLC [13]. EBC has traditionally been employed to assess inflammatory markers across a range of respiratory disorders, such as asthma, chronic obstructive pulmonary disease (COPD), idiopathic pulmonary fibrosis, cystic fibrosis, and obstructive sleep apnea. Recent studies have extended its use to the genomic analysis of driver mutations in EBC samples from NSCLC patients, enabled by advancements in genomic sequencing and molecular analysis [14, 15, 16, 17]. EBC offers a potentially complementary approach, as it directly samples respiratory tract secretions, potentially providing a more tumor‐proximal source of biomarkers in LC. Prior studies, such as those by Youssef et al. have established EBC's utility for mutation analysis in LC [15, 18]. Our study builds on this foundation by evaluating EBC's efficacy in detecting fusion transcripts alongside tissue and liquid biopsies, providing comparative insights into the diagnostic strengths and limitations of EBC in NSCLC. These findings indicate that EBC samples may provide a valuable, noninvasive, and repeatable tool for detecting molecular alterations in LC, enhancing both diagnostic and monitoring capabilities in NSCLC.

Chromosomal rearrangements leading to gene fusions play a critical role in the development of lung adenocarcinoma. Targeted therapies directed at fusion proteins resulting from *ALK*, *ROS1*, *RET*, and other gene fusions underscore the importance of routinely identifying these alterations in patients with lung adenocarcinoma. Fusion transcripts in oncogenic driver genes, such as *ALK*, *ROS1*, *RET*, and *NTRK1*, have become essential predictive biomarkers for lung tumors, necessitating advanced, minimally invasive technologies capable of detecting these changes from limited biopsy sample [5]. Traditionally, methods like fluorescence in situ hybridization (FISH), immunohistochemistry (IHC), and reverse transcription‐polymerase chain reaction (RT‐PCR) were used to detect LC fusions. However, the recent implementation of next‐generation RNA sequencing (RNA‐seq) allows for the simultaneous detection of gene fusion transcripts with high sensitivity and specificity [6]. Identifying targetable gene fusions with precision is crucial for guiding the choice of targeted therapies in lung adenocarcinoma, enhancing personalized treatment strategies.

The aim of this study is to detect fusion transcripts in the *ALK*, *RET*, *ROS1*, and *NTRK1* genes through next‐generation RNA‐seq on samples obtained from paraffin‐embedded tissue, liquid biopsy, and EBC from 30 lung adenocarcinoma patients undergoing treatment. Additionally, the study seeks to evaluate the efficacy of liquid biopsy and EBC samples in RNA isolation and fusion transcript detection, comparing these noninvasive methods to each other and to archival tissue samples collected at diagnosis, which serve as the gold standard. This comparison aims to establish the feasibility of using noninvasive techniques to guide appropriate treatment options for patients, potentially enhancing personalized treatment strategies in clinical oncology.

## 2 Materials and Methods

### 2.1 Patients

**FIGURE 1:**
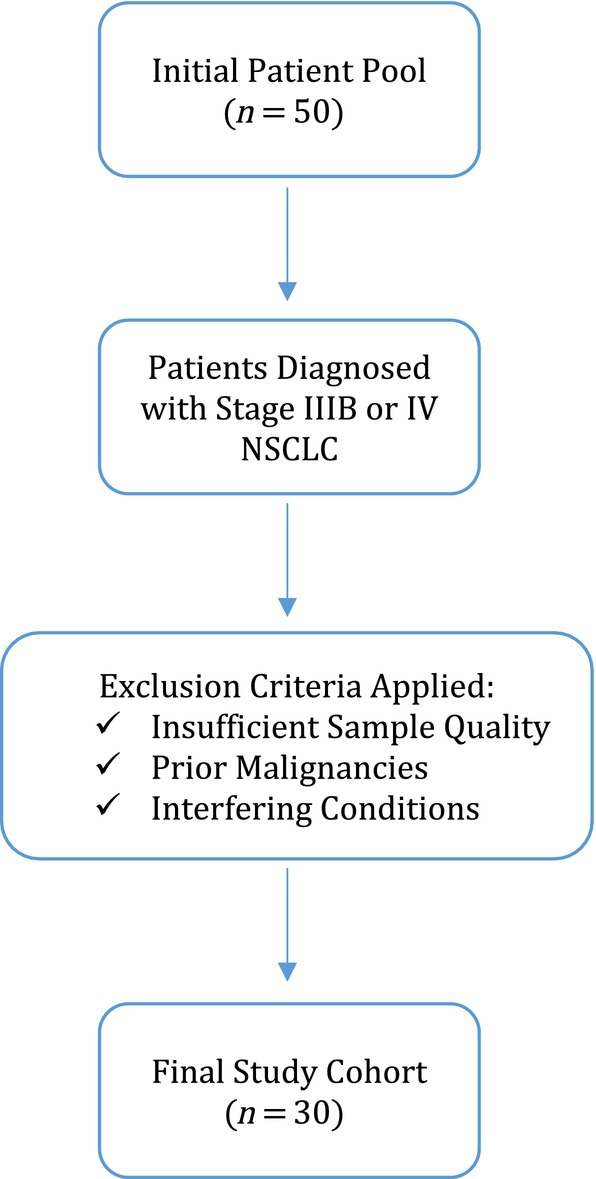
Patient flowchart illustrating inclusion and exclusion process.

This study included 30 patients with a confirmed diagnosis of primary lung adenocarcinoma (Stage IIIB or IV), with or without solitary or multiple synchronous metastases, who were followed at the Department of Chest Diseases, Ege University Medical School, between 2021 and 2023. The diagnosis was verified by the Department of Pathology at Ege University Medical School. A power analysis was conducted to determine the appropriate sample size for detecting clinically significant fusion transcripts in NSCLC patients at advanced stages (Stage IIIB or IV). Given the relatively low prevalence of certain targetable fusion transcripts (e.g., ALK, RET, ROS1, and NTRK1) in NSCLC, a sample size of 30 patients was selected to achieve adequate statistical power for detecting these alterations across various sample types (FFPE tissue, plasma, and EBC). This sample size was estimated to provide sufficient power (> 80%) to identify clinically relevant fusion events with a significance level (*α*) of 0.05. The chosen cohort size allows for the comparative evaluation of detection rates across sample types, ensuring robust analysis of EBC's diagnostic performance relative to conventional tissue and liquid biopsy methods. To enhance transparency and address potential selection bias, a patient flowchart (Figure 1) has been included, detailing the inclusion and exclusion process. Initial recruitment included all patients diagnosed with Stage IIIB or IV lung adenocarcinoma within the study period. Exclusion criteria were applied to ensure data quality and consistency, including cases with insufficient sample quantity or quality for RNA extraction, prior malignancies, or pre‐existing conditions that could interfere with the study's objectives. The flowchart provides a clear overview of patient progression from recruitment to final inclusion, improving transparency in data handling. Comprehensive demographic and clinical characteristics of each patient were recorded, ensuring detailed documentation for reproducibility. This study received approval from the Institutional Ethics Committee for Medical Research (Approval Number: 20‐9.1T/42), and all participants provided written informed consent prior to enrollment.

### 2.2 Collection of Clinical Samples

In this study, formalin‐fixed paraffin‐embedded (FFPE) tissue samples were obtained from archival blocks following lung adenocarcinoma diagnosis, with six 10‐μm sections cut from each block for analysis. To evaluate the efficacy of noninvasive diagnostic methods for detecting molecular biomarkers, additional samples, including EBC and peripheral blood, were collected from each patient.

For EBC collection, patients were instructed to refrain from smoking, eating, or drinking for at least 8 h before sampling. Immediately before collection, they rinsed their mouths with water to reduce potential contaminants. EBC samples were obtained by condensing exhaled breath with a custom‐built glass condenser maintained at −20°C. Patients exhaled into the condenser through a mouthpiece for 15 min, yielding 2–2.5 mL of EBC, which was immediately transferred into DNA‐ and RNA‐free tubes and stored at −20°C until analysis. To ensure sample purity, patients inhaled purified, humidified air through a nasal mask during the collection procedure.

Peripheral blood samples (5 mL) were collected in EDTA tubes and immediately centrifuged at 4000*g* for 10 min to isolate plasma, which was then stored at −80°C for subsequent analysis. Collecting these samples at diagnosis established a baseline, with archival FFPE samples serving as a reference standard for molecular and genetic comparisons across sample types. This approach allowed for a comprehensive evaluation of circulating biomarkers in both plasma and EBC samples [15].

### 2.3 Next‐Generation RNA‐Seq

Total RNA was extracted from FFPE samples using the GeneJET FFPE RNA Kit (Thermo Scientific), whereas cfRNA was isolated from patients' EBC and plasma samples using the PureLink RNA Mini Kit, following the respective manufacturer's protocols. RNA concentrations were initially measured with a Nanodrop 1000 spectrophotometer (Thermo Scientific). To ensure accurate quantification for next‐generation sequencing (NGS), RNA concentration was further verified using the Qubit RNA HS Assay Kit (Thermo Scientific), as per the manufacturer's instructions. Only RNA samples with concentrations exceeding 10 ng and acceptable purity ratios (260/280 and 260/230) of 1.8 or higher were included for sequencing analysis in this study. In alignment with the NCCN Guidelines Version 11.2024, which identify ALK rearrangements, ROS1 rearrangements, RET rearrangements, and NTRK1/2/3 gene fusions as clinically significant biomarkers in NSCLC, this study focused on the analysis of these gene alterations. All samples were subjected to NGS to detect the presence of these targeted gene rearrangements, ensuring adherence to the recommended biomarker testing outlined by the NCCN. By evaluating *ALK*, *ROS1*, *RET*, and *NTRK1/2/3* alterations, we aimed to provide comprehensive molecular profiling that could support tailored therapeutic strategies in NSCLC management [19].

### 2.4 cDNA Synthesis

Complementary DNA (cDNA) synthesis was performed using the SuperScript IV VILO Master Mix cDNA synthesis kit (Thermo Scientific), following the protocol provided by the manufacturer [20].

### 2.5 RNA‐Seq

The synthesized cDNA was analyzed using the Ion AmpliSeq RNA Fusion Lung Cancer Research Panel, designed to detect 85 clinically relevant fusion transcripts in the *ALK*, *RET*, *ROS1*, and *NTRK1* genes associated with LC. Targeted RNA‐seq was performed on the Ion Torrent Personal Genome Machine (PGM) system [21]. Fusion transcript presence was assessed in cfRNA isolated from plasma and EBC samples to monitor molecular alterations in lung tumor tissues during treatment.

### 2.6 Statistical Analysis

RNA extracted from FFPE tissue, EBC, and plasma samples collected from 30 patients with advanced‐stage lung adenocarcinoma was subjected to next‐generation RNA‐seq. The resulting BAM and BAI files from the Ion Torrent PGM system were converted to FASTQ format, and sequencing data were analyzed using the CLC Workbench bioinformatics platform. Sequence alignment was performed against the hg38 reference genome. Fusion transcripts with statistical significance (*p* < 0.05), identified through web‐based analysis software, were considered for further analysis. Comparative statistical analyses were conducted using GraphPad Prism version 10.5. All detected fusion transcripts were cross‐referenced with the COSMIC (Catalogue of Somatic Mutations in Cancer) [22] and FUSIONGDB2 [23] databases to confirm previously reported cancer‐associated gene rearrangements.

## 3 Results

The mean age of female patients was 62.1 ± 7.2 years, whereas male patients had a mean age of 61.3 ± 10.7 years. The overall mean age of the cohort was 61.5 ± 9.8 years, with an age range of 40 to 79 years. Among the cohort, six patients (2 females, 4 males) had single‐site metastasis, eight patients (2 females, 6 males) had multisite metastases, and 15 patients (4 females, 11 males) showed no metastasis. The mean treatment duration was 13.3 ± 10.4 days, with males averaging 14.8 ± 10.8 days and females averaging 9.2 ± 8.4 days, showing no statistically significant difference (*p* = 0.1979). Based on treatment modalities, three patients (1 female, 2 males) received monotherapy, 10 patients (1 female, 9 males) received a combination of chemotherapy and immunotherapy, 11 patients (3 females, 8 males) received chemotherapy and radiotherapy, and six patients (3 females, 3 males) received a combination of chemotherapy, radiotherapy, and immunotherapy (Table 1).

**TABLE 1:** Demographic and clinical characteristics of the cases.

		Adenocarcinoma, *n*: 30 (%100)
Gender	Female	8 (27.7)
	Male	22 (73.3)
Age (year)	Female	62.13 ± 7.16
	Male	63.32 ± 10.71
Stage	IIIB	8 (27.7)
	IV	22 (73.3)
Treatment	Monothreaphy	3 (10)
	CT, IT	10 (33.3)
	CT, RT	11 (36.7)
	CT, RT, IT	6 (20.0)
Metastasis	Detected	14 (46.7)
	Not detected	16 (53.3)

Abbreviations: CT, chemotherapy; IT, immunotherapy; RT, radiotherapy.

Next‐generation RNA‐seq of RNA isolated from clinical samples revealed that *ALK*, *ROS1*, *RET*, and *NTRK1* fusion transcripts were detectable in FFPE tissue, plasma, and EBC samples. Clinically significant fusions were identified in three cases: *KIF5B*‐*ALK* fusion was detected in both FFPE and EBC samples, *KIF5B*‐*RET* fusion was observed in both FFPE and plasma samples, and *SQSTM1*‐*ALK* fusion was found exclusively in the EBC sample, with each fusion representing 3.33% of cases.

**FIGURE 2:**
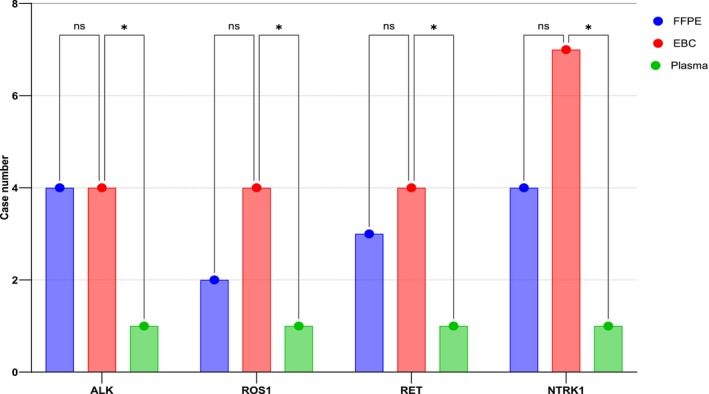
Correlation of *ALK*, *ROS1*, *RET*, and *NTRK1* fusions in clinical samples.

**FIGURE 3:**
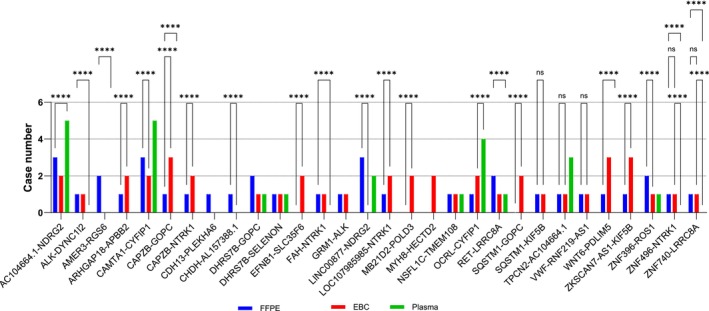
Fusion transcripts of unknown clinical significance detected in FFPE tissue, EBC, and plasma samples.

Further analysis revealed the presence of fusion transcripts in FFPE samples, with *ALK* and *NTRK1* fusions each detected in four cases, *RET* in three cases, and *ROS1* in two cases. In EBC samples, *ALK*, *ROS1*, and *RET* fusions were each detected in four cases, whereas *NTRK1* was found in seven cases. By contrast, plasma samples detected *ALK*, *ROS1*, *RET*, and *NTRK1* fusions in only one case each (Figure 2). These findings demonstrated a stronger correlation between tissue and EBC samples than with plasma samples for detecting *ALK*, *ROS1*, and *RET* fusion transcripts, suggesting that EBC could serve as a reliable noninvasive alternative to tissue biopsy for identifying these fusions. Fusion transcripts, including those of uncertain clinical significance, were detected in 70% of FFPE and EBC samples and in 50% of plasma samples (Figure 3).

Analysis of clinically significant fusion transcripts revealed notable correlations across different sample types. Specifically, the *KIF5B*‐*ALK* fusion was detected in both FFPE and EBC samples but was absent in plasma. In a separate case, the *KIF5B*‐*RET* fusion was identified in both FFPE and plasma samples. Additionally, the *SQSTM1*‐*ALK* fusion was uniquely detected in the EBC sample (Table 2).

**TABLE 2:** Clinically significant *ALK* and *RET* fusions detected in paraffin tissue, EBC, and plasma samples.

Fusion	FFPE	EBC	Plasma
*KIF5B*‐*ALK*	1	1	0
*KIF5B*‐*RET*	1	0	1
*SQSTM1*‐*ALK*	0	1	0

Due to the absence of observed treatment response or drug resistance during follow‐up in Stage IIIB and IV cases, we were unable to evaluate the relationship between detected fusion transcripts and treatment outcomes. Furthermore, as no progression events occurred among the 30 adenocarcinoma cases during the study period, it was not possible to assess associations between fusion transcripts and disease‐free or overall survival.

## 4 Discussion

In NSCLC, gene rearrangements involving *ALK*, *ROS1*, and *RET* occur in approximately 3%–7%, 1%–2%, and 1%–2% of cases, respectively, while *NTRK* fusions are rarer, occurring in less than 1% of cases [24, 25, 26, 27, 28, 29, 30]. In Turkey, *ALK* rearrangements in NSCLC are reported to range from 3.4% to 8.3%, and *ROS1* rearrangements from 0.4% to 1.9%, with *RET* and *NTRK* rearrangements being even less frequent [2]. Over 90 distinct *ALK* fusion partners have been identified, including genes such as *KIF5B*, *KLC1*, *TFG*, *TPR*, *HIP1*, *STRN*, *DCTN1*, *SQSTM1*, *NPM1*, *BCL11A*, and *BIRC6*. These *ALK* fusions result in abnormal kinase activity, which drives cancer cell proliferation through pathways like RAS–ERK and PI3K‐AKT. The development of *ALK* inhibitors has significantly improved survival outcomes in advanced NSCLC patients with *ALK* fusions, with response rates exceeding 80% and 5‐year survival rates surpassing 60% [24].

Although tissue and liquid biopsies are routinely employed for detecting, monitoring, and guiding treatment based on fusion transcripts, no prior studies have demonstrated the detection of specific fusion transcripts in EBC samples. This study highlights the potential of incorporating multiple sample types, including EBC, into cancer diagnostics. According to NCCN guidelines, tissue biopsy remains the gold standard for molecular testing, with liquid biopsy recommended when re‐biopsy is unfeasible or tissue samples are inadequate. Our findings indicate that RNA from EBC, in conjunction with tissue and liquid biopsies, can detect clinically significant and novel fusion transcripts via NGS analysis. This positions EBC as a promising noninvasive source for NSCLC diagnostics and monitoring. The detection of novel fusion transcripts in FFPE, plasma, and EBC samples underscores EBC's potential in identifying previously uncharacterized fusions, thereby enhancing diagnostic capabilities and advancing cancer research (Figure 3).

Our study identified clinically significant fusion transcripts in FFPE, EBC, and plasma samples from adenocarcinoma patients. Notably, the *KIF5B*‐*ALK* fusion (COSF1381), a clinically important fusion in LC, was detected in both FFPE and EBC samples from the same patient. This fusion, involving the *KIF5B* and *ALK* genes, creates an oncogenic kinase that activates downstream pathways, such as *STAT3*, promoting tumor growth, migration, and invasion [31]. Accurate identification of *ALK* fusion variants is essential for identifying NSCLC patients likely to benefit from *ALK* inhibitor therapy; for example, *KIF5B*‐*ALK*–positive NSCLC patients often respond favorably to *ALK* TKIs, including crizotinib [31, 32]. Additionally, we detected the *SQSTM1*‐*ALK* fusion exclusively in the EBC sample, highlighting a relatively rare but clinically significant variant in NSCLC, seen in approximately 1.1% of cases, in contrast to the more common *EML4*‐*ALK* fusions, which represent about 81.5% of all *ALK* fusions. These findings suggest that EBC samples could serve as a valuable tool in molecular diagnosis and support treatment planning in LC patients.

*RET* fusions are a rare but distinct molecular subtype of NSCLC, found in approximately 1%–2% of cases. Patients with *RET* fusion–positive NSCLC often present with unique clinical characteristics, such as a higher incidence of brain metastases, which occur in roughly 46% of these patients. *RET* rearrangements are more frequently observed in younger, nonsmoking patients with adenocarcinoma histology and often advanced disease. While further research is necessary to elucidate the clinical significance of specific *RET* fusion partners, current evidence indicates that the efficacy of selective *RET* inhibitors may vary depending on the fusion partner. Retrospective studies suggest that *RET* fusion–positive NSCLC patients receiving *RET* inhibitor therapy have improved survival outcomes compared to those without targeted treatment [33]. In our study, the *KIF5B*‐*RET* fusion (COSF1232) was detected in both FFPE and plasma samples from one patient but was absent in the EBC sample, potentially due to tumor heterogeneity, where genetic variations across different tumor regions may lead to the absence of specific fusions in certain samples. Notably, EBC samples demonstrated higher efficacy in detecting *NTRK1* fusions, indicating that EBC may serve as a valuable noninvasive tool for monitoring fusion transcripts in LC.

Advances in RNA‐seq technologies are continually uncovering previously unrecognized fusion events in solid tumors, including LC. Our RNA‐seq analysis identified 30 fusion transcripts with uncertain clinical implications across tissue, liquid biopsy, and EBC samples, indicating that these transcripts may contribute to NSCLC pathogenesis or represent potential therapeutic targets. Further studies are needed to elucidate the biological functions of these fusion transcripts and determine their relevance in LC.

In conclusion, EBC sampling offers significant advantages over traditional biopsies, particularly for longitudinal monitoring of tumor dynamics in LC. The capacity of EBC to detect and analyze specific biomarkers, such as fusion transcripts, holds promise for facilitating personalized treatment strategies and improving patient outcomes. Future studies should prioritize the validation of EBC–based assays and the optimization of their sensitivity and specificity to support integration into routine clinical practice. Additionally, large‐scale clinical trials are warranted to establish standardized protocols and comprehensively evaluate EBC's role in LC management.

### 4.1 Limitations

This study has certain limitations that may introduce bias. First, the single‐institution design may result in selection bias, as our findings reflect the characteristics of a specific patient population and may not be fully generalizable to other settings. To address this, we recommend future research include multi‐institutional cohorts to improve the external validity and applicability of the findings.

Additionally, a patient flowchart was not included in the original manuscript, which may limit the transparency of our selection process. In this revised version, we have included a patient flowchart detailing the recruitment, inclusion, and exclusion process, allowing for a clearer understanding of the study population and enhancing the reproducibility of the study.

Another potential source of bias is measurement variability in RNA quality from EBC samples. Due to the absence of a standardized EBC collection protocol, differences in collection techniques may have affected RNA quality. We have detailed the EBC collection procedure used in this study to clarify our methodology. However, to minimize measurement bias in future studies, we recommend the development of standardized protocols for EBC collection, which could improve consistency in RNA quality across studies.

## 5 Author Contributions

Aslı Tetik Vardarlı and Tuncay Goksel were involved in the conception and design. Tuncay Goksel, Hakan Soydaner Karakus, Ali Veral, Fusun Pelit, Su Ozgur and Korcan Korba collected data. Aslı Tetik Vardarlı and Cumhur Gunduz drafted the manuscript. Aslı Tetik Vardarlı and Burcu Boluk performed the RNA seq analysis. Aslı Tetik Vardarlı and Su Ozgur conducted the literature search. All authors contributed to the article and approved the submitted version. All authors agree to be accountable for all aspects of the work.

## Conflicts of Interest

The authors declare no conflicts of interest.

## Data Availability

The data supporting the findings of this study, including detailed characterization of fusion transcripts detected in formalin‐fixed paraffin‐embedded (FFPE) tissue, plasma, and exhaled breath condensate (EBC) samples, are available in the Supporting Information. Supporting Information is available at Thoracic Cancer online.
